# Biofunctionalized Materials Featuring Feedforward and Feedback Circuits Exemplified by the Detection of Botulinum Toxin A

**DOI:** 10.1002/advs.201801320

**Published:** 2018-11-28

**Authors:** Hanna J. Wagner, Svenja Kemmer, Raphael Engesser, Jens Timmer, Wilfried Weber

**Affiliations:** ^1^ Faculty of Biology University of Freiburg Schänzlestraße 1 79104 Freiburg Germany; ^2^ BIOSS—Centre for Biological Signalling Studies University of Freiburg Schänzlestraße 18 79104 Freiburg Germany; ^3^ Spemann Graduate School of Biology and Medicine (SGBM) University of Freiburg Albertstraße 19a 79104 Freiburg Germany; ^4^ Institute of Physics University of Freiburg Hermann‐Herder Straße 3 79104 Freiburg Germany

**Keywords:** biohybrid, information processing, mathematical modeling, proteases, synthetic biology

## Abstract

Feedforward and feedback loops are key regulatory elements in cellular signaling and information processing. Synthetic biology exploits these elements for the design of molecular circuits that enable the reprogramming and control of specific cellular functions. These circuits serve as a basis for the engineering of complex cellular networks, opening the door for numerous medical and biotechnological applications. Here, a similar principle is applied. Feedforward and positive feedback circuits are incorporated into biohybrid polymer materials in order to develop signal‐sensing and signal‐processing devices. This concept is exemplified by the detection of the proteolytic activity of the botulinum neurotoxin A. To this aim, site‐specific proteases are incorporated into receiver, transmitter, and output materials, and their release, diffusion, and/or activation are wired according to a feedforward or a positive feedback circuit. The development of a quantitative mathematical model enables analysis and comparison of the performance of both systems. The flexible design could be easily adapted to detect other toxins or molecules of interest. Furthermore, cellular signaling or gene regulatory pathways could provide additional blueprints for the development of novel biohybrid circuits. Such information‐processing, material‐embedded biological circuits hold great promise for a variety of analytical, medical, or biotechnological applications.

## 1 Introduction

In order to react to changes in their environment, living organisms must perceive and process specific signals. On cellular level, receptors sense input signals and transduce this information to intracellular signaling pathways that initiate specific cellular responses. These pathways involve the amplification of the input signal by means of second messengers and enzymatic cascades. Most signaling pathways are characterized by complex feedforward and feedback regulation and crosstalk with other pathways, thus forming intricate signaling networks.^1^, ^2^, ^3^, ^4^ Over the past decade, the field of synthetic biology has harnessed the deepened understanding of signaling pathways and networks to design and engineer novel, synthetic signaling pathways and circuits that can reprogram cellular functions.^5^, ^6^ Cells capable of performing Boolean logic operations,^7^, ^8^ counting the number of input signals,^9^ or detecting borders between cell populations^10^ have been engineered based on circuits of activators, repressors, feedback elements, and feedforward elements.^11^, ^12^ This synthetic biological concept has delivered new approaches for studying signaling mechanisms, and opened the door for diverse applications in many fields including biotechnology and medicine.^13^ The synthetic biological principle is not only limited to the programming of cells, but has also recently been exploited for the development of interconnected biohybrid materials with information‐processing capabilities.^14^, ^15^ For this, synthetic biological receptors, enzymes, and switches conferred sensor, transmitter, and actuator functionalities to polymer materials to make the systems count light pulses or to amplify input signals.

Inspired by principles of cellular signal transduction and synthetic biology, we embedded biological signaling motifs into polymer materials and interconnected them to information‐sensing and processing circuits. We exemplified this concept by a positive feedforward and a positive feedback topology. These circuit designs are fundamental motifs in signal transduction pathways^16^, ^17^, ^18^ and are harnessed for the programming of synthetic biological circuits.^19^, ^20^, ^21^ Here, we show that, similar to cellular systems, the polymer material‐embedded signaling circuits were able to sense and process a specific input signal and produce a quantitative output. We demonstrated the functionality of either circuit design by the detection of the proteolytic activity of the botulinum neurotoxin A light chain (BoNT/A‐LC). To analyze the effect of both topologies on the system behavior and identify differences in kinetics and dynamic range, we developed mathematical models calibrated on experimental data.

This work illustrates that the integration of signaling motifs into polymer materials can yield biohybrid circuits capable of processing input signals according to feedforward and/or feedback‐based circuit topologies. Based on the versatility of the underlying design principle, we propose that synthetic biology and cell signaling provide a rich source of potential blueprints for conferring computational functions and diverse input/output modalities to biofunctionalized materials. Such devices that autonomously sense and process information could have a variety of promising applications in analytics, regenerative medicine, or drug delivery.^22^

## 2 Results and Discussion

### 2.1 Design of Polymer Material‐Embedded Feedforward and Feedback Circuits

**Figure 1:**
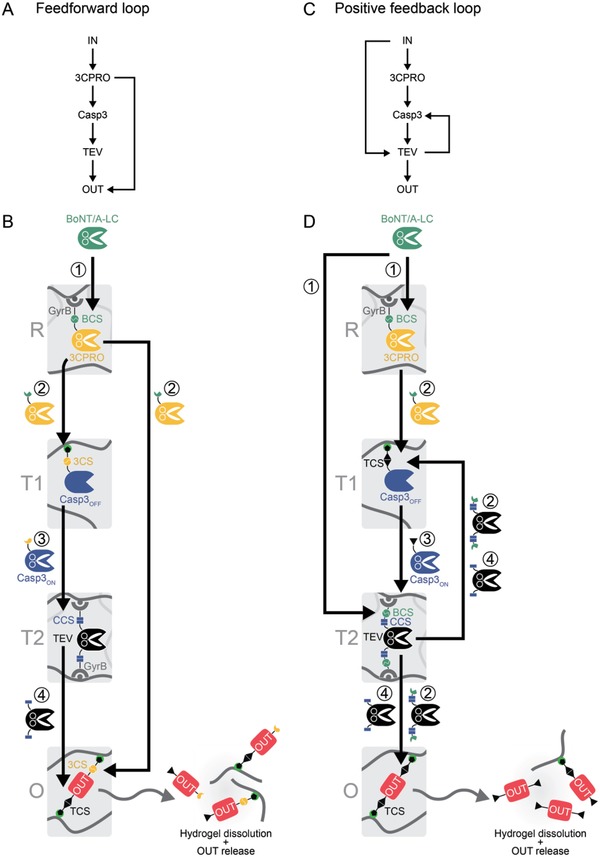
Design of biological circuits incorporated into polymer materials. A) Enzymatic feedforward circuit diagram. The input signal (IN) triggers a proteolytic feedforward circuit that responds by the release of an output signal (OUT). B) Incorporation of the enzymatic feedforward circuit into polymer materials. The input (BoNT/A‐LC)‐sensing receiver material (module R) is composed of 3C protease (3CPRO) that is bound to novobiocin‐functionalized crosslinked agarose (indicated by gray lines) via the bacterial gyrase subunit B (GyrB). The linker region between 3CPRO and GyrB contains a BoNT/A‐LC cleavage site (BCS). The transmitter module T1 contains 3CPRO‐activatable caspase‐3 (Casp3_OFF_) bound to crosslinked, Ni^2+^–nitrilotriacetic acid (Ni–NTA)‐functionalized polyacrylamide (indicated by the gray lines) via a 3CPRO cleavage site (3CS)‐containing His‐tag (black pentagon) anchor. In module T2, tobacco etch virus protease (TEV) is bound via two Casp3‐cleavable (CCS, Casp3 cleavage site) GyrB anchors to novobiocin‐functionalized agarose. The output material (module O) is composed of linear Ni–NTA‐functionalized polyacrylamide (gray lines) crosslinked to a hydrogel by the mCherry output protein (OUT) containing an N‐terminal TEV‐cleavable (TCS, TEV cleavage site) and a C‐terminal 3CPRO‐cleavable His‐tag linker. In the presence of BoNT/A‐LC (1), 3CPRO is released (2) and induces a proteolytic cascade by cleaving and activating Casp3_OFF_ (3), which in turn cleaves the CCSs in module T2 thus triggering the release of TEV (4). Released TEV reaches its target sites in module O and cleaves the crosslinking protein resulting in the dissolution of the hydrogel and the release of OUT. Additionally, 3CPRO cleaves its target sites in module O, thus supporting the dissolution of module O (positive feedforward loop). C) Positive feedback loop circuit diagram. A proteolytic circuit with feedforward and positive feedback loops senses IN and responds by releasing OUT. D) Incorporation of the combined feedforward and feedback circuit into polymer materials. Compared to the feedforward system (panels (A) and (B)), the following minor changes were made to modules T1, T2, and O: the two linkers between TEV and GyrB contain not only CCS but also BCS; the linkers of Casp3_OFF_ and OUT contain only TCS and no 3CS. In the presence of BoNT/A‐LC (1), 3CPRO and TEV are released from modules R and T2, respectively (2). 3CPRO activates Casp3_OFF_ and TEV triggers the release of Casp3 (3) and cleaves the crosslinking protein, thus initiating the dissolution of the hydrogel and the release of OUT. Activated Casp_ON_ releases additional TEV (4), which enhances the dissolution of the hydrogel and the release of OUT (positive feedback loop).

To develop polymer material‐embedded circuits for the sensing and processing of input signals, we evaluated two design motifs inspired by information‐processing circuits in cellular signal transduction^1^, ^23^, ^24^ and synthetic biology:^25^ one design was based on a positive feedforward loop (**Figure** **1**A,B) the other on a positive feedback loop (Figure 1C,D).

We exemplified the design concept by the detection of the *Clostridium botulinum* BoNT/A‐LC. BoNT/A‐LC is a zinc‐dependent metalloprotease and specifically cleaves and inactivates soluble *N*‐ethylmaleimide‐sensitive factor attachment protein receptor (SNARE) complex proteins.^26^ We engineered proteolytic cascades that can be activated by the input (BoNT/A‐LC) and release a quantitative output. They involved the human rhinovirus‐14 3C protease (3CPRO), human caspase‐3 (Casp3), and the tobacco etch virus protease (TEV). We incorporated the enzymatic cascades into polymer materials by designing four modules interconnected in a positive feedforward loop (Figure 1B) or in a positive feedback loop (Figure 1D) configuration. The individual modules act as receiver (R), transmitters (T1 and T2), or output (O) (Figure 1B,D). To integrate the protein building blocks into the polymer materials, we used the noncovalent interaction between bacterial gyrase subunit B (GyrB) and novobiocin or between a hexahistidine‐tag (His‐tag) and Ni^2+^–nitrilotriacetic acid (Ni–NTA).

Modules R and T2 are composed of novobiocin‐functionalized crosslinked agarose for the binding of GyrB‐fusion proteins. Modules T1 and O are synthesized from linear Ni–NTA‐functionalized polyacrylamide (poly(AAm‐*co*‐Ni–NTA–AAm)) that can be physically crosslinked to form a hydrogel by double‐His‐tagged proteins.^27^, ^28^

In the feedforward configuration (Figure 1B), module R contains 3CPRO fused to GyrB via a BoNT/A‐LC cleavage site (BCS). In material T1 an inactive, 3CPRO‐activatable version of Casp3 (Casp3_OFF_) is bound via a 3CPRO cleavage site (3CS) to the polymer. Module T2 contains TEV bound to the material via two Casp3 cleavage site (CCS)‐containing GyrB anchors. Module O is composed of the output (OUT, mCherry red fluorescent protein) crosslinking the Ni–NTA polymer via an N‐terminal TEV cleavage site (TCS)‐containing and a C‐terminal 3CS‐containing His‐tag anchor. In the presence of BoNT/A‐LC (1), 3CPRO is released from module R (2) and mediates the release, cleavage, and activation of Casp3_OFF_ (3) for the induction of a proteolytic cascade. Casp3_ON_ cleaves its target sites in module T2 and triggers the release of TEV (4). TEV cleaves the crosslinking output protein and thus induces the dissolution of the material and the release of OUT. Concurrently, 3CPRO directly cleaves the crosslinking protein in module O (feedforward loop) and thus enhances hydrogel dissolution and the release of OUT.

The feedback system amplifies the input signal in a combined positive feedforward and positive feedback loop configuration (Figure 1C). Here, BoNT/A‐LC directly triggers the release of TEV (feedforward wiring), and released TEV both induces the dissolution of material O and, together with 3CPRO, releases and activates Casp3_OFF_, which in turn enhances the release of TEV and thus the dissolution of the material O (positive feedback loop). The mode of function of the corresponding material system is depicted in Figure 1D. Module R has the same composition as module R of the feedforward system. In modules T1 and O, the linkers of 3CPRO‐activatable Casp3_OFF_ and OUT contain TCSs instead of 3CSs. To enable both BoNT/A‐LC and Casp3_ON_‐mediated release of TEV from module T2, TEV is linked to the module via two linkers that both contain a CCS and a BCS. BoNT/A‐LC (1) triggers the release of 3CPRO and TEV from modules R and T2, respectively (2). TEV cleaves the hydrogel crosslinker and releases Casp3_OFF_. Free Casp3_OFF_ is activated by 3CPRO (3) and mediates the release of additional TEV from module T2 (4, feedback loop), thus enhancing the dissolution of module O and the concomitant release of the output protein.

After designing the feedforward and the feedback systems, we built and tested all modules individually, as described in the following sections. Since modules T1 and O do not interact, we reduced the complexity of the systems by integrating both modules into the same material. In a second step, we assembled the systems, established mathematical models for the two configurations, and calibrated them with experimental data to estimate kinetic parameters for both systems. We used these model‐derived parameter estimates for comparative analysis of the functionality of both systems over a range of BoNT/A‐LC input concentrations.

### 2.2 Synthesis and Testing of the Receiver Module R

**Figure 2:**
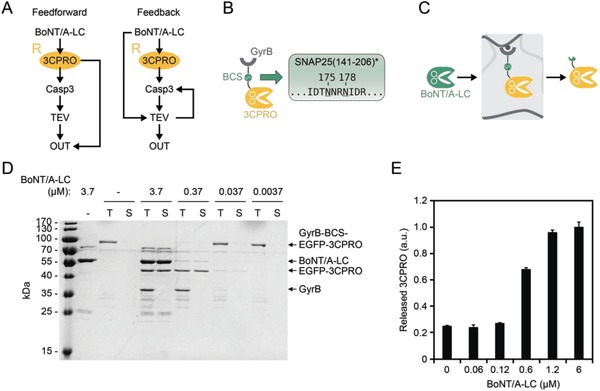
Synthesis and testing of the input‐sensing module R. A) Module R senses the input BoNT/A‐LC and responds by activation of an enzymatic cascade and an additional feedforward or positive feedback loop. B) Design of the protein sensor. 3C protease (3CPRO) is fused to the bacterial gyrase subunit B (GyrB) via EGFP (to simplify the schematics, EGFP is not shown) and a BoNT/A‐LC cleavage site (BCS)‐containing linker. The BCS is composed of amino acid residues 141–206 of the SNAP25 protein modified containing two glutamine‐to‐asparagine exchanges at amino acid positions 175 and 178 (designated SNAP25(141–206)*), which prevents nonspecific cleavage by 3CPRO. C) For characterization of module R, the material was incubated with BoNT/A‐LC and the release of 3CPRO was determined. D) SDS‐PAGE analysis of BoNT/A‐LC‐induced 3CPRO release. The material (1.6 mg Sepharose per sample) was incubated with the indicated concentrations of BoNT/A‐LC, and the cleavage products in the supernatant (S) or total sample (T, supernatant and material‐bound protein) were evaluated. E) Quantification of released 3CPRO. Module A (0.5 mg Sepharose per sample) was incubated with the indicated concentrations of BoNT/A‐LC, and the release of 3CPRO was determined by measuring the fluorescence of EGFP fused to 3CPRO. Values were normalized to the fluorescence of 3CPRO released with the highest concentration of BoNT/A‐LC (6 × 10^−6^ m). Mean values ± s.e.m. of four replicates are shown.

The receiver module R is present in both the feedforward and the positive feedback system (**Figure** **2**A). In both systems, it comprises 3CPRO that is bound via a BoNT/A‐LC cleavable GyrB anchor to novobiocin‐functionalized crosslinked agarose (Figure 2B,C). We used amino acids 141–206 of the SNARE protein SNAP25 as BCS and added a TEV‐removable N‐terminal His‐tag. Interestingly, it was not possible to purify this construct via Ni–NTA affinity chromatography (Figure S1A, Supporting Information). We observed overexpressed protein in the bacterial lysate corresponding to separated GyrB and 3CPRO. In contrast, a control construct composed of His‐tagged GyrB–enhanced green fluorescent protein (EGFP)–3CPRO and lacking any protease cleavage site was well expressed and easily purified. When we omitted the TCS from our receiver construct, we obtained a protein fragment corresponding to His‐tagged GyrB in the eluate (Figure S1B, Supporting Information). This was in accordance with a previous report of nonspecific cleavage of the TCS by 3CPRO.^29^ Nevertheless, the construct was processed between GyrB and 3CPRO, most likely by another unspecific cleavage site of 3CPRO in the BCS. Native cleavage sites of 3CPRO contain glutamine at the P1 position and glycine at the P1′ position. We mutated the glutamine in SNAP25(141–206) at position 198 to asparagine (Q198N), but still did not obtain purified full‐length protein. In contrast, a Q175N and Q178N double mutant (referred to as SNAP25(141–206)*, Figure 2B) was well expressed and easily purified in the full‐length form (Figure S1C, Supporting Information). To confirm that BoNT/A‐LC is still able to cleave SNAP25(141–206)* and to release 3CPRO from module R (Figure 1C), we incubated the material with different concentrations of BoNT/A‐LC and evaluated the cleavage products by sodium dodecyl sulfate–polyacrylamide gel electrophoresis (SDS‐PAGE). Analysis of the material supernatant (S) and total protein (T, supernatant and material‐bound protein) revealed BoNT/A‐LC‐dependent cleavage and release of 3CPRO (Figure 2D). Furthermore, we quantified 3CPRO in the supernatant, which confirmed a BoNT/A‐LC concentration‐dependent release of 3CPRO (Figure 2E). These results indicate that the double mutated SNAP25(141–206)* site is not processed by 3CPRO and can still be specifically cleaved by active BoNT/A‐LC.

### 2.3 Synthesis and Testing of the Transmitter Module T2

**Figure 3:**
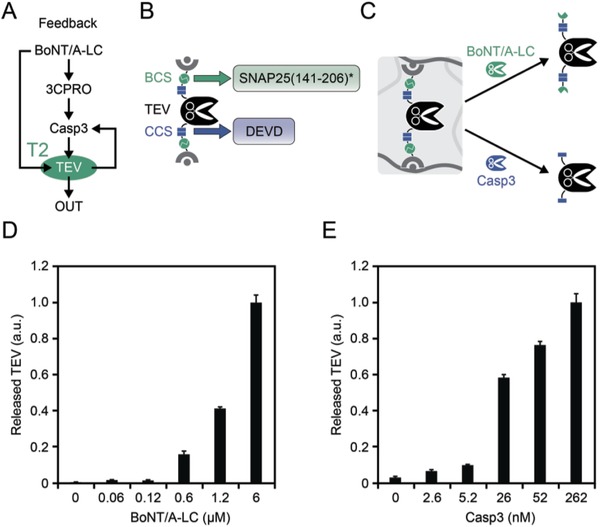
Design and characterization of module T2. A) Module T2 of the feedback system directly processes the BoNT/A‐LC input and responds to Casp3. B) Design of the TEV construct incorporated in module T2. TEV is fused on both sides to the bacterial gyrase subunit B (GyrB) by linkers containing cleavage sites for BoNT/A‐LC (BCS) and Casp3 (CCS). To prevent unintended cleavage by 3CPRO, BCS is composed of mutated SNAP25 cleavage site (designated SNAP(141–206)*). C) In the presence of BoNT/A‐LC or Casp3, TEV is released from the material. D) BoNT/A‐LC‐mediated release of TEV. The material (1.6 mg Sepharose per sample) was incubated with the indicated concentrations of BoNT/A‐LC and released TEV was quantified by determining its activity in the supernatant. Values were normalized to the activity obtained with the highest concentration of BoNT/A‐LC (6 × 10^−6^ m). E) Casp3‐mediated release of TEV. The experimental procedure was the same as described in panel (D). Values were normalized to the activity obtained with 262 × 10^−9^ m Casp3. Mean values ± s.e.m. of four replicates are shown.

Module T2 of the feedforward and feedback systems is composed of TEV bound to novobiocin‐functionalized crosslinked agarose by two Casp3‐cleavable GyrB anchors. Module T2 of the feedback system additionally contains BCSs (SNAP25(141–206)*) in both linker regions (**Figure** **3**A,B). Therefore, TEV can be released either by BoNT/A‐LC or by Casp3 (Figure 3C). Analysis of TEV activity in the supernatant of the material confirmed BoNT/A‐LC and Casp3 concentration‐dependent cleavage and release of TEV (Figure 3D,E).

### 2.4 Synthesis and Testing of the Hydrogel Featuring the Transmitter Module T1 and the Output Module O

**Figure 4:**
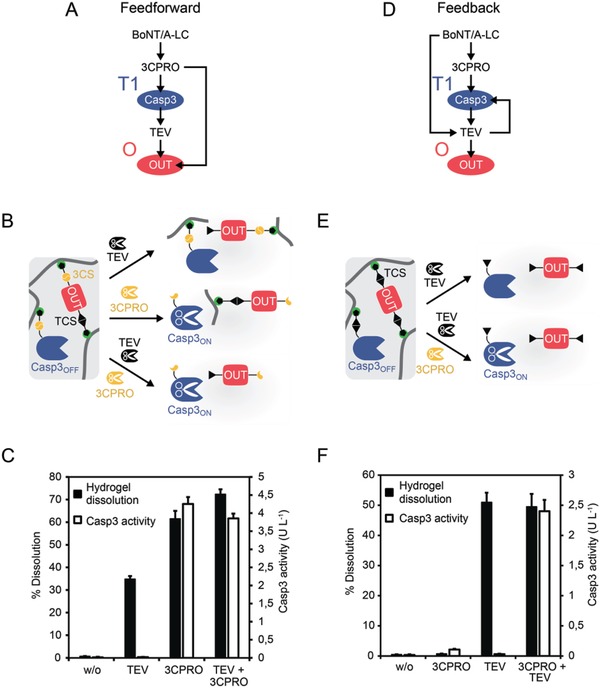
Characterization of the hybrid hydrogel harboring modules T1 and O. A) In the feedforward system, the hydrogel contains Casp3_OFF_, which is activated and released by 3CPRO, and the output protein (OUT), which responds to both 3CPRO and TEV. B) In the hydrogel of the feedforward system, OUT crosslinks the polymers via TCS‐ and 3CS‐containing His‐linkers (module O). Additionally, 3CPRO‐activatable Casp3_OFF_ was incorporated via a 3CPRO‐cleavable His‐linker (module T1). C) Experimental validation of the functionality of the hybrid T1/O material of the feedforward system. The dissolution of the material and the release of active Casp3_ON_ were determined in the absence or in the presence of TEV and/or 3CPRO. Hydrogels containing 0.02 U anchored Casp3_OFF_ were incubated with the indicated proteases (1 × 10^−6^ m TEV and/or 10 × 10^−6^ m 3CPRO). After 18 h, the dissolution of the material and the activity of released Casp3_ON_ were determined. Mean values ± s.e.m. of six replicates are shown. D) The hydrogel of the feedback system responds to TEV by triggering the release of OUT and Casp3_OFF_. E) In the feedback system, OUT crosslinks the polymers via two TEV‐cleavable His‐anchors. Casp3_OFF_ is incorporated via a TEV‐cleavable linker. In the presence of TEV, Casp3_OFF_ and OUT are released, which is accompanied by the dissolution of the material. Addition of 3CPRO proteolytically activates Casp3_OFF_. F) Experimental validation of the functionality of the hybrid T1/O material of the feedback system. Hydrogels were synthesized with 0.02 U anchored Casp3_OFF_ and incubated with 0.6 × 10^−6^ m 3CPRO and/or TEV for 18 h. The dissolution of the material and the release of active Casp3_ON_ were determined. Mean values ± s.e.m. of six replicates are shown.

Modules T1 and O were constructed within the same material. This hydrogel comprised poly(AAm‐*co*‐Ni–NTA–AAm) crosslinked via N‐ and C‐terminal His‐tags of OUT and also contained the 3CPRO‐inducible Casp3_OFF_. In the feedforward system, the OUT construct contains a TCS in the N‐terminal linker region and a 3CS in the C‐terminal linker region (**Figure** **4**A,B). Although 3CPRO is, in principle, able to cleave at TCS, we have previously shown that the processing of a TCS‐containing hydrogel crosslinker occurs at negligible rates (see Figure 2 in ref. 30). The 3CPRO‐inducible Casp3_OFF_ constituting the module T1 component of the feedforward hydrogel was incorporated via a 3CS‐containing His‐tag anchor. We tested modules T1 and O of the feedforward system by adding TEV and/or 3CPRO to the hydrogel and measuring the release of Casp3_ON_ and OUT, which correlates with hydrogel dissolution (Figure 4B). In the presence of TEV, the N‐terminal linker of the crosslinking OUT protein is cleaved, leading to the dissolution of the gel and the release of polymer‐bound Casp3_OFF_ (Figure 4B,C). If 3CPRO is added (with or without TEV) to the feedforward hydrogel, the C‐terminal linkers of OUT and Casp3_OFF_ are cleaved, resulting in the dissolution of the gel and the release active Casp3_ON_ (Figure 4C).

Modules T1 and O of the positive feedback system (Figure 4D,E) were generated as described previously.^14^, ^30^ Here, both linker regions of the crosslinking OUT protein contain a TCS, and the 3CPRO‐inducible Casp3_OFF_ is incorporated via a TEV‐cleavable TCS linker (Figure 4E). In the presence of TEV, the crosslinking OUT protein is cleaved, leading to the dissolution of the hydrogel and the release of OUT and Casp3_OFF_ (Figure 4F). 3CPRO alone is neither able to dissolve the gel, nor to release active Casp3_ON_ (Figure 4F). Only the presence of both proteases results in the dissolution of the material and release of active Casp3_ON_ (Figure 4F). Noteworthy, the dissolution of module O and release of OUT were readily detectable by eye (Figure S2, Supporting Information).

### 2.5 Assembly of the Feedforward‐ and Feedback‐Based Biohybrid Circuits

After confirming the functionality of each module, we assembled the complete signal‐processing systems. We used modules R and T2 corresponding to enzyme activities of 0.15 mU 3CPRO and 0.15 relative units (RU) TEV for the feedforward system and to 0.0375 mU 3CPRO and 0.3 RU TEV for the feedback system. The differences in applied 3CPRO and TEV amounts for both systems are reflected by the differences in the number of 3CSs and TCSs in the feedforward (two 3CSs in Casp3_OFF_, one in OUT; one TCS in OUT) and the feedback (one 3CS for Casp3_OFF_ activation; three TCSs in Casp3_OFF_ and OUT) system. We combined modules R and T2 with 25 µL T1/O hydrogels containing 0.01 U Casp3 in a total volume of 1.5 mL.

**Figure 5:**
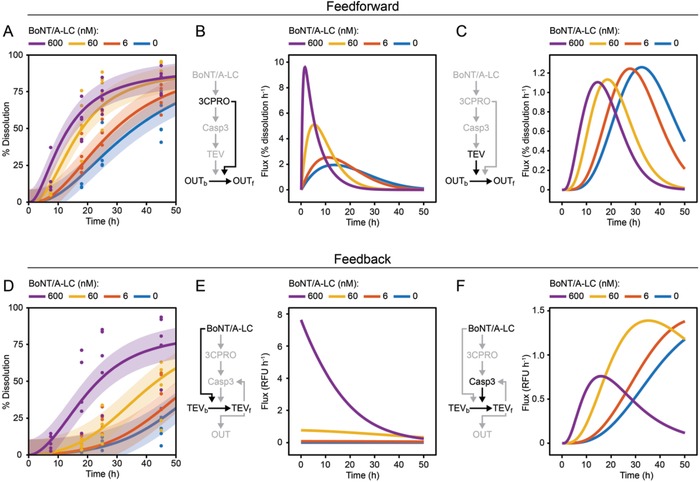
Mathematical model‐based analysis of the assembled feedforward and feedback systems. A) Time course of the response of the feedforward system at different BoNT/A‐LC concentrations. The system was assembled with 0.15 mU 3CPRO, 0.01 U Casp3, and 0.15 RU TEV in modules R, T1, and T2, respectively, and incubated with the indicated concentrations of BoNT/A‐LC. The dissolution of module O was monitored by quantifying the release of OUT (mCherry). The experimental data are indicated by dots, and the model fits by curves. The shaded bands correspond to one standard deviation. B,C) Model‐based flux analysis of the feedforward system. The conversion of bound OUT (OUT_b_) to free OUT (OUT_f_) directly catalyzed by 3CPRO through the feedforward loop (panel B)—see reactions v9 and v11 in Figure S3 in the Supporting Information—and the additional impact of the enzymatic cascade represented by the TEV‐mediated release of OUT (panel C)—see reactions v8 and v10 in Figure S3 in the Supporting Information—were evaluated for the indicated BoNT/A‐LC concentrations. D) Time course of the response of the feedback system at different BoNT/A‐LC concentrations. The system was assembled with 0.0375 mU 3CPRO, 0.01 U Casp3, and 0.3 RU TEV in modules R, T1, and T2, respectively. The indicated concentrations of BoNT/A‐LC were added and the dissolution of module O was monitored by quantifying released OUT (mCherry). The experimental data are indicated by dots, and the model fits by curves. The shaded bands correspond to one standard deviation. E,F) Model‐based flux analysis of the feedback system. The conversion of bound TEV (TEV_b_) to free TEV (TEV_f_) directly catalyzed by BoNT/A‐LC through the feedforward loop (panel E)—see reaction v8 in Figure S4 in the Supporting Information—and the feedback‐dependent release of TEV catalyzed by Casp3_ON_ (panel F)–see reaction v6 in Figure S4 in the Supporting Information—were analyzed for the indicated BoNT/A‐LC concentrations. Fluxes of the feedback system are given in relative fluorescence units (RFU) TEV per hour.

**Figure 6:**
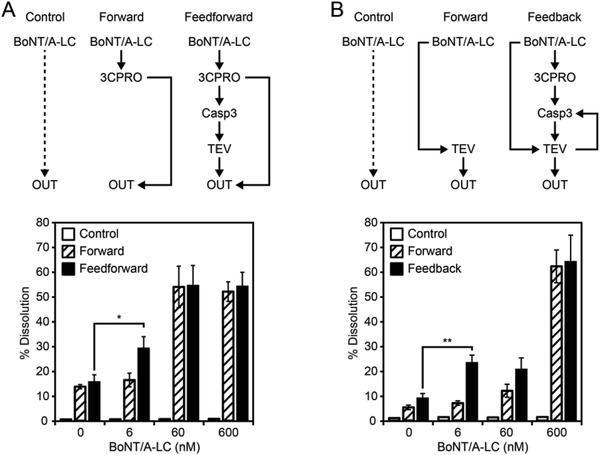
Experimental analysis of the assembled feedforward and feedback system. A) Comparison of the sole feedforward loop (forward) with the complete feedforward system (feedforward). The system was assembled with 0.15 mU 3CPRO, 0.15 RU TEV, and 0.01 U Casp3 (complete system, top right), or without module T1 (Casp3) to prevent TEV‐mediated dissolution of module O (sole forward pathway, top middle). A setup without modules R, T1, and T2 served as control (control, top left). The indicated concentrations of BoNT/A‐LC were added, and the dissolution of module O of the control (white bars), the sole forward system (hatched bars), and the complete feedforward system (black bars) was determined after 18 h by quantifying released OUT (mCherry). Mean values ± s.e.m. of five replicates are shown. B) Comparison of the sole feedforward loop (forward pathway) with the complete feedback system. The system was assembled with 0.0375 mU 3CPRO, 0.3 RU TEV, and 0.01 U Casp3 (complete system, top right), or without module T1 (Casp3) to disrupt the positive feedback loop (forward system, top middle). The control configuration lacked modules R, T1, and T2 (control, top left). The indicated concentrations of BoNT/A‐LC were added and the dissolution of module O of the control (white bars), the forward system (white bars), and the complete feedback system (black bars) was determined after 25 h by quantifying released OUT (mCherry). Mean values ± s.e.m. of five replicates are shown.

We added different BoNT/A‐LC input concentrations and monitored the dissolution of the OUT hydrogel (**Figure** **5**A,D). Both systems responded to BoNT/A‐LC in a concentration‐dependent manner, but with different kinetics. The feedforward system displayed faster dissolution of material O (Figure 5A vs Figure 5D). In contrast, the feedback system showed a higher dynamic range (Figure 5A vs Figure 5D). To analyze the systems in more detail, we developed quantitative mathematical models based on ordinary differential equations (ODEs) for both systems and calibrated them with experimental data of the individual modules (Figures 2, 3, 4; Figure 3b in ref. 14) and the complete systems (Figure 5A,D; see Figures S3–S6 and the derivation of the models in the Supporting Information). The uncertainties and the identifiability of parameters were analyzed using an approach based on the profile likelihood.^31^ This analysis showed that all parameters are identifiable and an eightfold crossvalidation confirmed the accuracy of the model (Figure S7, Supporting Information). The model fits are indicated by the curves in Figure 5A,D. To analyze the contributions of the individual reaction pathways within the systems, we conducted a model‐based flux analysis. At high BoNT/A‐LC concentrations, the dominant flux regarding the release of OUT in the feedforward system is the conversion of bound OUT to free OUT directly catalyzed by 3CPRO through the feedforward loop (Figure 5B). In the feedback system, the feedforward and the feedback loop are integrated on the level of the TEV protease. Here, the dominant flux for the conversion of bound TEV to released TEV is directly catalyzed by BoNT/A‐LC via the feedforward loop (Figure 5E). In contrast, at low BoNT/A‐LC concentrations, the flux via the 3CPRO‐mediated dissolution in the feedforward system is reduced to similar levels as the flux catalyzed by TEV (Figure 5B,C). Similarly, the flux induced by the positive feedback via Casp3 in the feedback system becomes dominant at low BoNT/A‐LC input concentrations (Figure 5E,F). Consequently, combining the enzyme cascade with the additional feedforward or feedback loops in the two configurations confers higher sensitivity to the systems. This is reflected in the experimental data (**Figure** **6**). We assembled the systems without Casp3 (T1), so that the dissolution of module O could be controlled solely via the feedforward loops, thus converting the systems into direct, forward configurations (hereafter, referred to as forward pathways). Comparison with the complete systems showed that the sole forward pathways were not able to distinguish between the absence and presence of the lowest applied BoNT/A‐LC concentration (6 × 10^−9^ m, hatched bars in Figure 6A,B). Moreover, BoNT/A‐LC was not able to induce any output response in the absence of receiver and transmitter modules (white bars in Figure 6A,B). In contrast, the complete systems were able to detect the lowest applied BoNT/A‐LC concentration (black bars in Figure 6A,B). Furthermore, the feedback system showed a dynamic range of sevenfold, which was twice as high as the dynamic range of the feedforward system (Figure 6A,B).

These results demonstrate that cell‐signaling motifs, synthetic biological tools, and polymer materials can be combined to construct biosensing devices, as exemplified by the detection of BoNT/A‐LC. The incorporation of biological building blocks into polymer materials enabled the modular assembly of biohybrid circuits featuring positive feedforward and positive feedback loop motifs. The differences in the response profiles obtained for the two configurations (feedforward vs feedback system) suggest the possibility of tuning the kinetics and dynamic range of the systems. Furthermore, these designs are highly flexible and can be adjusted by exchanging protease cleavage sites, or by using affinity pairs whose association or dissociation can be controlled by small molecules or light.^15^, ^28^, ^32^

## 3 Conclusions

In this study, we apply molecular tools and concepts derived from cellular signaling and synthetic biology to design and construct polymer materials with embedded biohybrid circuits featuring positive feedforward and feedback motifs. Both circuit topologies have crucial roles in the processing and amplification of signals in both endogenous and synthetic signaling pathways. Interestingly, we obtained different response profiles for the two circuit configurations, suggesting the possibility of customizing the system performance by adjusting the circuit topology. Both circuits were able to sense and process the lowest applied input concentrations of BoNT/A‐LC. However, the circuit with a positive feedback loop displayed improved performance; in that it had a greater dynamic range. On account of the flexible designs of the systems, they could be easily adapted for the detection of other input signals such as toxins or pathogen‐derived proteases. The BoNT/A‐LC cleavage sites, for example, could more generally be exchanged with those of other BoNT serotypes, of other toxins such as the tetanus toxin, or of viral proteases. Furthermore, synthetic biology offers a variety of affinity pairs whose dissociation can be triggered by competitive ligands. Such molecular switches could be incorporated into polymer materials^27^, ^28^, ^32^, ^33^ and thus complement the protease‐based response elements presented herein.

The development of biofunctionalized materials with embedded biological circuits has been gaining increasing interest. This new field exploits the synergies of cellular signaling, synthetic biology, and polymer materials, and opens the door to advanced and creative systems in various fields. Cellular signaling and synthetic biology exhibit diverse circuit motifs that could serve as blueprints for the development of novel information‐processing biohybrid devices. Furthermore, the large multitude of other synthetic biological tools and signaling mechanisms could be exploited to endow biohybrid circuits with additional functions. As presented here, such systems hold promise as analytical biosensors. Moreover, they could be adapted for the release of diverse biomolecules, including biocatalysts or therapeutic molecules and could thus offer novel solutions in the biotechnological and medical sectors.

## 4 Experimental Section

*Plasmids*: The cloning of all plasmids used in this study is described in Tables S2 and S3 (Supporting Information).

*Protein Production and Purification*: All recombinant proteins were produced in *Escherichia coli* BL21(DE3)pLysS. Bacteria were grown at 37 °C with shaking (150 rpm) in 1 L lysogeny broth (LB) medium supplemented with ampicillin (100 µg mL^−1^) and chloramphenicol (36 µg mL^−1^). At an OD_600_ of 0.6, protein production was induced with 1 × 10^−3^ m isopropyl β‐d‐1‐thiogalactopyranoside (IPTG). The hydrogel crosslinking OUT protein (HJW2 and HJW261) and Casp3 (HJW181 and HJW182) were produced at 37 °C for 4 h. TEV (HJW199 and HJW265) was produced at 30 °C for 4 h, and BoNT/A‐LC (HJW144) was produced at 25 °C for 20 h. Bacteria were collected by centrifugation at 6000 × *g* for 10 min, resuspended in 35 mL Ni Lysis buffer (50 × 10^−3^ m NaH_2_PO_4_, 300 × 10^−3^ m NaCl, and 10 × 10^−3^ m imidazole, pH 8.0) and disrupted by sonication (Bandelin Sonoplus HD 3100 homogenizer, 60% amplitude and 0.5 s/1 s pulse/pause intervals). Cellular debris was removed by centrifugation for 30 min at 30 000 × *g* and 4 °C, and proteins were purified by gravity‐flow Ni–NTA affinity chromatography. Ni–NTA agarose (QIAGEN, Cat.‐No. 30230; 2 mL bed volume) was equilibrated with 15 mL Ni Lysis buffer. After loading the cleared lysate, the column was washed twice with 30 mL Ni Wash buffer (50 × 10^−3^ m NaH_2_PO_4_, 300 × 10^−3^ m NaCl, and 20 × 10^−3^ m imidazole, pH 8.0). The protein was eluted with Ni elution buffer (50 × 10^−3^ m NaH_2_PO_4_, 300 × 10^−3^ m NaCl, and 250 × 10^−3^ m imidazole, pH 8.0). TEV and Casp3 protein constructs were supplemented with 10 × 10^−3^ m 2‐mercaptoethanol (2‐ME). The crosslinking proteins (HJW2 and HJW261) and BoNT/A‐LC were dialyzed twice against 5 L Ni Lysis buffer w/o imidazole (50 × 10^−3^ m NaH_2_PO_4_ and 300 × 10^−3^ m NaCl, pH 8.0) at 4 °C, stirring, using SnakeSkin dialysis tubing (3.5k molecular weight cut‐off (MWCO), Fisher Scientific, Cat.‐No. 10005743). The buffer of Casp3 constructs was exchanged with Ni Lysis buffer without imidazole supplemented with 10 × 10^−3^ m 2‐ME (hereafter, referred to as hydrogel buffer) using a dextran desalting column (5k MWCO, 5 mL, Thermo Fisher, Cat.‐No. 43230).

*Synthesis of Modules R and T2*: Novobiocin‐functionalized crosslinked agarose was prepared with a degree of functionalization of 0.17 nmol g^−1^ of epoxy‐activated Sepharose, as previously described.^14^, ^30^ Briefly, for the functionalization of 1 g material, 0.88 mmol novobiocin was dissolved in 4.4 mL 0.3 m sodium carbonate–bicarbonate buffer, pH 9.5, mixed with 1 g epoxy‐activated Sepharose 6B (GE Healthcare, Cat.‐No. 17048001), and incubated at 37 °C for 16 h with agitation. Nonreacted epoxy‐groups were blocked by incubation with 1 m ethanolamine (pH 8.5) for 4 h at 37 °C with agitation. The material was washed with alternating washing steps of water, buffer A (100 × 10^−3^ m Tris‐HCl, 500 × 10^−3^ m NaCl, pH 8), water, and buffer B (100 × 10^−3^ m acetate buffer, 500 × 10^−3^ m NaCl, pH 4). The novobiocin‐functionalized Sepharose was blocked in phosphate‐buffered saline (PBS) supplemented with 1% (w/v) bovine serum albumin (BSA, Sigma‐Aldrich, Cat‐No. A6003) with agitation at 4 °C.

GyrB‐containing proteins were bound to the material by incubating the novobiocin‐functionalized agarose with a fivefold molar excess of protein in modified Ni Elution buffer (supplemented with 10 × 10^−3^ m 2‐ME and 0.1% (w/v) BSA, and in the case of TEV with 10% (v/v) glycerol) overnight at 4 °C with agitation. Unbound protein was removed by washing with hydrogel buffer supplemented with 0.1% (w/v) BSA and 10% (v/v) glycerol.

*Synthesis of the Hydrogel with the Components of Modules T1 and O*: For the synthesis of hydrogels, the crosslinking OUT proteins (HJW2 or HJW261) were concentrated using Spin‐X UF 6 concentrators with 10k MWCO (Corning, Cat.‐No. 431488). 15 µL crosslinking protein (50 mg mL^−1^) supplemented with 0.01 U Casp3 (unless indicated otherwise) was mixed with 10 µL poly(AAm‐*co*‐Ni–NTA–AAm) (generated in a previous study^14^, ^30^; 2% (w/v) in hydrogel buffer) on a siliconized glass slide (Sigma‐Aldrich, Cat.‐No. SL2). After incubation in a humidified atmosphere overnight at room temperature (RT), the gels were transferred to 1 mL hydrogel buffer. After 6 h of incubation at RT, the gels were transferred to fresh hydrogel buffer and incubated at RT overnight.

*Assembly of the Feedforward and Feedback Systems*: For the assembly of the complete systems, an equal amount of unbound novobiocin‐functionalized Sepharose was added to modules R and T2 to minimize unintended dissociation of the GyrB‐functionalized proteins from these materials. After incubation at RT for 30 min with agitation, the proteolytic activities of the TEV‐ and 3CPRO‐materials were determined (see “Analytical Methods”). The TEV‐ and 3CPRO‐containing Sepharose modules were combined with one T1/O hybrid hydrogel in 1.5 mL hydrogel buffer. The indicated amounts of BoNT/A‐LC were added and the dissolution of the hydrogels was monitored (see “Analytical Methods”).

*Analytical Methods*: Protein purity and sizes were evaluated by SDS‐PAGE (12% (w/v) gels) and subsequent Coomassie staining. Protein concentrations were determined by Bradford assay (Bio‐Rad, Cat.‐No. 5000006).

The release of 3CPRO from material module A was determined by measuring the fluorescence of EGFP at 490/520 nm Ex/Em.

The activity of 3CPRO was determined using the HRV 3C Protease Activity Assay Kit (BioVision, Cat.‐No. K214‐100). 50 µL of 3CPRO was mixed with 2.5 µL of the substrate and the absorbance at 405 nm was monitored for at least 20 min.

To determine the activity of 3CPRO‐inducible Casp3_OFF_ for hydrogel synthesis, its proteolytic activity needed to be induced prior to the activity measurement (Casp3_ON_). For this, 5 µg of 3CPRO (HJW4) was added to 15 µg of Casp3_OFF_ in a total volume of 50 µL hydrogel buffer. After incubation at RT overnight, the sample was diluted 1:100 in hydrogel buffer. 30 µL of Casp3_ON_ sample was mixed with 30 µL of 2X reaction buffer and 3 µL of 4 × 10^−3^ m aspartate‐glutamate‐valine‐aspartate (DEVD)‐*p*‐nitroaniline substrate, and the increase in absorbance was monitored for at least 20 min. The activity of Casp3_ON_ released from hydrogels was determined by using nondiluted samples of the material supernatants without further 3CPRO treatment.

For Casp3 and 3CPRO, a dilution series of *p*‐nitroaniline ((0–500) × 10^−6^ m) served as calibration standard. 1 U Casp3 or 3CPRO corresponded to the amount of protease that processed 1 µmol substrate min^−1^ under assay conditions.

The proteolytic activity of TEV was determined using the SensoLyte 520 TEV Activity Assay Kit (AnaSpec, Cat.‐No. 72227), as previously described.^14^ Briefly, 25 µL of the TEV sample was mixed with 25 µL of substrate solution and the fluorescence signal was measured at 490/520 nm Ex/Em. The obtained signals were correlated to a 5‐carboxyfluorescein (5‐FAM) dilution series. 1 RU TEV corresponded to the amount of protease that cleaved the amount of substrate equivalent to 1 pmol 5‐FAM min^−1^ under assay conditions.

T1/O hybrid hydrogel dissolution was monitored by determining the fluorescence of released output protein (mCherry) in black 96‐well plates (Corning, Cat.‐No. 3915) at 575/620 nm Ex/Em using an Infinite M200 pro microplate reader (Tecan). At the end of each experiment, the gels were fully dissolved with 25 × 10^−3^ m ethylenediaminetetraacetic acid (EDTA). The fluorescence obtained from fully dissolved gels corresponded to 100% dissolution and was used for normalization.

*Statistics*: Mean values of at least four replicates were shown with error bars representing ± standard error of the mean (s.e.m.). Statistical significance was assessed by unpaired *t*‐test using GraphPad Prism 5; *p < 0.05, **p < 0.005.

## Conflict of Interest

The authors declare no conflict of interest.

## Supporting information

Supplementary
