# Supplementary material for: Biofunctionalized Materials Featuring Feedforward and Feedback Circuits Exemplified by the Detection of Botulinum Toxin A
Source: Adv Sci (Weinh). 2018 Nov 28;6(4):1801320. doi: 10.1002/advs.201801320 (PMC6382303; doi:10.1002/advs.201801320)
Supplement: Supplementary file 1 — Supplementary [file ADVS-6-1801320-s001.pdf]

Copyright WILEY-VCH Verlag GmbH & Co. KGaA, 69469 Weinheim, Germany, 2018.

ADVANCED SCIENCE Open Access

Supporting Information

for *Adv. Sci.,* DOI: 10.1002/advs.201801320

Biofunctionalized Materials Featuring Feedforward and Feedback Circuits Exemplified by the Detection of Botulinum Toxin A

*Hanna J. Wagner, Svenja Kemmer, Raphael Engesser, Jens Timmer, and Wilfried Weber**

WILEY-VCH

Copyright WILEY-VCH Verlag GmbH & Co. KGaA, 69469 Weinheim, Germany, 2018.

Supporting Information

Biofunctionalized Materials Featuring Feedforward and Feedback Circuits Exemplified by the Detection of Botulinum Toxin A

*Hanna J. Wagner, Svenja Kemmer, Raphael Engesser, Jens Timmer and Wilfried Weber**

*Correspondence: wilfried.weber@biologie.uni-freiburg.de

Contents

Suppl. Information S1. Mathematical Modeling 2
Figure S1. Design of the 3CPRO construct of module R. 7
Figure S2. Photo of the output module O. 7
Figure S3. Mathematical model of the feedforward system. 9
Figure S4. Mathematical model of the feedback system. 11
Figure S5. Characterization of the feedforward system. 12
Figure S6. Characterization of the feedback system. 13
Figure S7. Profile likelihood and cross validations of estimated parameters. 14
Table S1. Parameters of the feedforward and the feedback system. 15
Table S2. Plasmids used in this study. 16
Table S3. Primers used for cloning of the plasmids in Table S2. 18
References 19

Suppl. Information S1. Mathematical Modeling

1. Derivation of the mathematical model

Mathematical models have been widely used to mechanistically understand and predict the performance of biochemical reaction networks. In the following we model the biomaterials-based detection systems as biochemical reaction networks incorporating all causal dependencies of the network structure. We use non-linear ordinary differential equations (ODEs) to describe the dynamic behavior of relevant system components termed states. Enzymatic reactions are modeled via Michaelis-Menten kinetics, whereas basal activation and deactivation rates are implemented as mass action. The unknown model parameters are estimated from experimental data using a maximum likelihood approach. To determine parameter uncertainties in terms of parameter confidence intervals and thus the identifiability of the system we analyze the profile likelihood for each parameter. In the following, the two biomaterials-based detection circuits describing a feedforward (Figure 1A and B) and an integrated feedforward-feedback (Figure 1C and D) system are derived.

The model scheme of the feedforward system is shown in Figure S2A. For the sake of simplicity, anchors of released proteins are not shown. After the first contact with an active chain of botulinum toxin A, 3C protease is released from its polymer and triggers a linear reaction cascade finally resulting in the cleavage of one anchor of the output protein mCherry (v8, v10). Its second anchor is directly cleaved by the 3C protease (v9, v11) resulting in a feedforward loop which enhances the systems response. The model consists of 13 reactions with corresponding fluxes v1-v13 (Figure S2B). Note that Michaelis-Menten terms are re-parameterized to facilitate fitting of enzymatic reactions in their linear range in relation to the substrate. The km_x parameters we used for modeling therefore represent the inverse of the original Michaelis-Menten constants Km_x. Given these fluxes the resulting set of ODEs can be formulated (Figure S2C) to describe the dynamic behavior of the system.

The structure of the integrated feedforward-feedback detection circuit, referred to as feedback system, is outlined in Figure S3A. In contrast to the feedforward system, it is not the 3C protease but the active chain of botulinum toxin A that directly triggers the feedforward loop. Here, the signal is already integrated on the level of the TEV protease that is immobilized with two anchors, each containing cleavage sites for botulinum toxin A and Caspase 3. Moreover, a positive feedback loop is integrated in the system as the release of Caspase 3 is triggered by active TEV. This model consists of ten equations with corresponding fluxes v1-v10 (Figure S3B), resulting in the ODE system depicted in Figure S3C. Note that the release of Caspase 3 and mCherry are both catalyzed by TEV protease and are modeled with the same rate constants for simplification.

2. Likelihood-based parameter estimation and identifiability analysis

In order to build up a mathematical model accurately describing an observed biochemical network, unknown model parameters have to be determined. It is often difficult to directly measure these parameters, e.g., kinetic rates. However, they can be estimated from experimental data by maximum likelihood estimation.

The ODE system of our two models can be described with the following vectorized scheme

$$\frac{d}{dt}\vec{x}(t) = \vec{f}\,(\vec{x}(t), \vec{p}). \quad (2.1)$$

Internal states are described as $\vec{x}(t)$ with initial concentrations $\vec{x}(0)$ and depend on unknown dynamic parameters $\vec{p}$, e.g. biochemical rates. Internal states are linked to experimental data by an observation function g

$$y(t) = g(\vec{x}(t), \vec{s}) + \varepsilon(t), \quad (2.2)$$

which depends on observation parameters $\vec{s}$, e.g. offset and scaling parameters. In addition, we assume a constant Gaussian error $\varepsilon(t) \sim N(0, \sigma^2)$ as measurement error, whose variance is estimated simultaneously with the dynamic parameters. The maximum likelihood estimation is performed as described in [1] in detail.

The resulting maximum likelihood estimate is further analyzed to assess uncertainty and identifiability of the optimal parameters. We therefor use the profile likelihood method [2] yielding confidence intervals for each parameter that are subsequently used for model reduction as described in [3].

3. Implementation of single experiments

Four different experiments were used to calibrate the models. In the following their implementation is described and corresponding observation functions are defined. All initial states are set to zero if not stated otherwise.

Experiment 1: Characterization of the feedforward system

In this experiment the biomaterials-based feedforward system outlined in Figure S2 was implemented to measure the relative amount of the output protein mCherry (OUT) under different concentrations of 3C protease, Caspase 3, TEV protease and botulinum toxin A. Thus,

the initial concentrations of $3CPRO_{bound}$, $Casp3_{bound}$ and TEV_{bound} were set to the applied concentrations and initials of all other states to zero. The starting value of OUT_{bound} was set to

$$[OUT_{bound}](0) = init_{OUT,FW}. \tag{3.4}$$

As observation function we used

$$OUT_{obs} = [OUT]. \tag{3.5}$$

The measurement error was modeled with a constant Gaussian error with the standard deviation $sd_{OUT\_obs,FW}$. Experimental data and model fits are shown in Figure S4A-H.

Experiment 2: Characterization of the feedback system

In this experiment the biomaterials-based feedback system outlined in Figure S3 was implemented to measure the relative amount of OUT again under different concentrations of 3C protease, Caspase 3, TEV protease and botulinum toxin A. Thus, the initial concentrations of $3CPRO_{bound}$, $Casp3_{bound}$ and TEV_{bound} were set to the applied concentrations and initials of all other states to zero. The starting value of OUT_{bound} was set to

$$[OUT_{bound}](0) = init_{OUT,FB}. \tag{3.5}$$

As observation function we used

$$OUT_{obs} = [OUT]. \tag{3.6}$$

The measurement error was modeled with a constant Gaussian error with the standard deviation $sd_{OUT\_obs,FB}$. The experimental data and model fits are shown in Figure S5.

Experiment 3: Characterization of the transmitter module T2

In this experiment the T2 module of the biomaterials-based feedback system was implemented to measure the release of TEV protease by different doses of Caspase 3 after four hours. The initial concentration of $Casp3_{active}$ was set to the different used concentrations. As applied in the experiment $init_{TEV\_bound}$ was set to 500 RU and initial concentrations of $3CPRO_{bound}$, $Casp3_{bound}$ and OUT_{bound} were set to zero. The following observation function was used

$$TEV_{obs_{DR}} = scale_{Exp100} \cdot [TEV]. \tag{3.1}$$

The measurement error was modeled with a constant Gaussian error with the standard deviation $sd_{TEV\_obs,DR}$. Experimental data are shown in Figure 3E

Experiment 4: Characterization of the output module O

In this experiment the output module O of the biomaterials-based feedback system was implemented to measure the release of OUT by different doses of TEV protease after 0, 6, 10 and 20 hours. The initial concentration of TEV was set to the different applied concentrations and initial concentrations of $3CPRO_{bound}$, $Casp3_{bound}$ and $Casp3_{active}$ were set to zero. The starting value of OUT_{bound} was set to

$$[OUT_{bound}](0) = init_{OUT,FW}. \tag{3.2}$$

The following observation function was used

$$OUT\_obs_{DR} = scale_{Exp15} \cdot [mCherry]. \tag{3.3}$$

The measurement error was again modeled with a constant Gaussian error with the standard deviation $sd_{OUT\_obs,DR}$. The experimental data are taken from Figure 3b in [1].

Standard deviations as well as initial values for OUT_{bound} differing from zero were estimated simultaneously with the dynamic parameters.

3. Fitting process and results

The feedforward and the feedback system have partly overlapping components and catalyzed reactions. Therefore, the two mathematical models share some of the parameters and fitting of the models to the experimental data was performed simultaneously. In total 29 parameters were fitted, including 21 dynamic parameters, two initial parameters, two scaling parameters and four error parameters. The set of estimated parameters is shown in Table S1.

Compilation, numerical integration, fitting and optimization of the model was performed with the MATLAB based free available software Data2Dynamics [4]. For the numerical integration of the ODE system we used the CVODES [5] solver and optimization was performed with the trust region based algorithm LSQNONLIN implemented in MATLAB [6]. The fitting was performed in logarithmic parameter space to scan the parameters over orders of magnitude. Moreover, multiple optimization runs with random sampled initial parameter sets were performed to ensure global convergence of the optimizer. Out of total 100 fits more than 80 % converged to the same lowest minimum indicating that the global optimum was found. The resulting model curves and data are shown in Figures S4 and S5. Shaded bands correspond to the estimated standard deviation of the Gaussian error model.

The identifiability analysis with the profile likelihood method showed that all inverse Michaelis-Menten parameters (km_x), except for $km_{Casp3activation\_3CPRO}$ that was fixed to the value $1.70 \cdot 10^{+00}$ as determined in [1], were practical non-identifiability towards zero. As introduced in [3] this is a case for model reduction. Thus, affected km values were set to zero for the following analysis resulting in linear reactions. It turned out that all other parameters are identifiable (Figure S6). 95 % point-wise confidence intervals calculated with the profile likelihood method are listed in Table 1 reaching from σ^- to σ^+.

4. Model validation

In order to test the model for robustness an eight-fold cross validation was performed. The 64 different experimental conditions in the data, excluding the single module characterization (Exp. 1 and 2), were randomly distributed into eight groups of 8 conditions. Seven data groups were used for parameter estimation to predict the dynamics of the eighth group. All predicted trajectories were describing the unfitted data very accurately with almost no visible difference to the originally fitted curves. Estimated parameter values of these eight optimization runs with reduced number of data conditions are indicated by colored stars in Figure S6. More than 95 % of all estimates are within the 95 % confidence interval of the original estimate calculated with the full data set indicating a robust model structure.

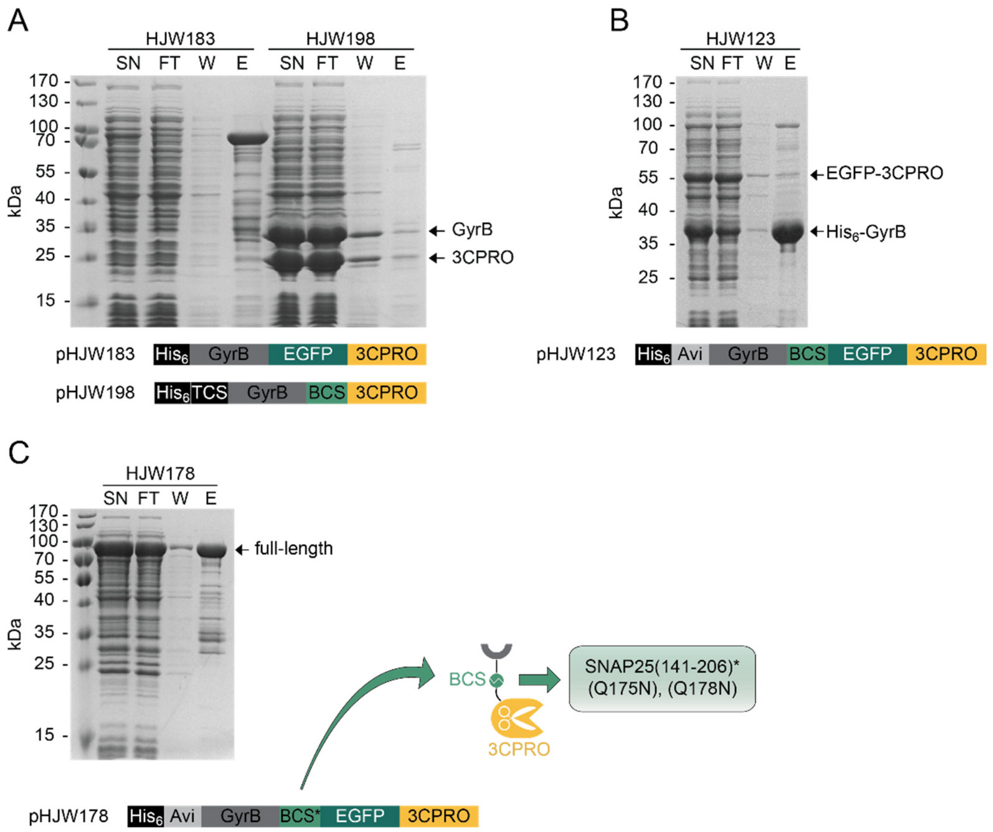

Figure S1. Design of the 3CPRO construct of module R. A) Expression and purification of the indicated constructs HJW183 and HJW198. Proteins were expressed in *E. coli* BL21(DE3)pLysS, purified via Ni-NTA affinity chromatography and analyzed via SDS-PAGE. B) SDS-PAGE of the expression and purification of a 3CPRO construct (HJW123) whose His-tag cannot be removed via a TCS. C) Expression and purification of a 3CPRO construct (HJW178) containing the indicated mutations in BCS. SN, supernatant; FT, flow-through; W, wash; E, elution; GyrB, bacterial gyrase subunit B; EGFP, enhanced green fluorescent protein; TCS, TEV cleavage site; BCS, amino acids 141-206 of SNAP25 (BoNT/A-LC cleavage site); Avi, Avi-tag; BCS*, mutated BCS.

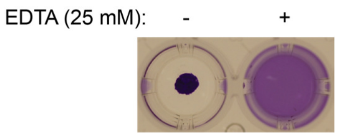

Figure S2. Photo of the output module O. The hydrogel was incubated in the presence (25 mM) or absence of EDTA in a total volume of 1.5 mL.

A

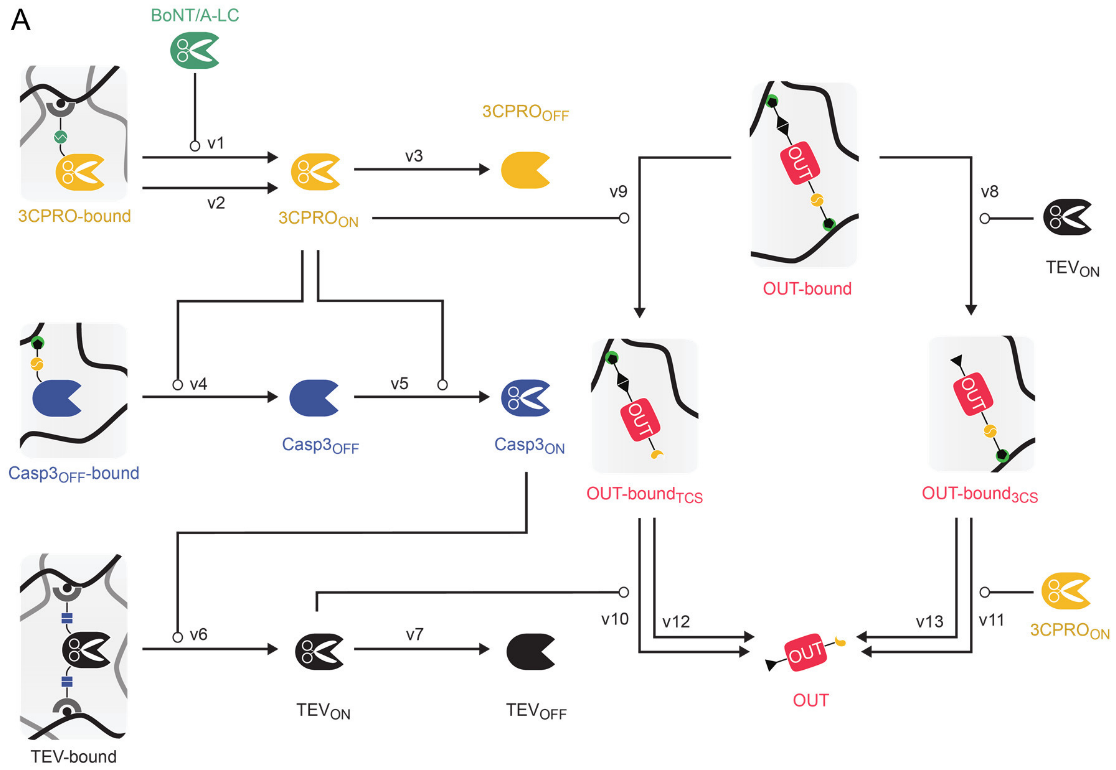

B

$$v_1 = k_{3CPROrelease,BoNT} \cdot [BoNT] \frac{[3CPRO_{bound}]}{1 + km_{3CPROrelease,\,BoNT} \cdot [3CPRO_{bound}]}$$

$$v_2 = k_{3CPROrelease,basal} \cdot [3CPRO_{bound}]$$

$$v_3 = k_{3CPROdeactivation} \cdot [3CPRO]$$

$$v_4 = k_{Casp3release,\,3CPRO,\,FW} \cdot [3CPRO] \frac{[Casp3_{bound}]}{1 + km_{Casp3release,\,3CPRO,\,FW} \cdot [Casp3_{bound}]}$$

$$v_5 = k_{Casp3activation,\,3CPRO} \cdot [3CPRO] \frac{[Casp3]}{1 + km_{Casp3activation,\,3CPRO} \cdot [Casp3]}$$

$$v_6 = k_{TEVrelease,\,CASP} \cdot [Casp3_{active}] \frac{[TEV_{bound}]}{1 + km_{TEVrelease,\,CASP} \cdot [TEV_{bound}]}$$

$$v_7 = k_{TEVdeactivation} \cdot [TEV]$$

$$v_8 = k_{OUTrelease,\,TEV,\,FW} \cdot [TEV] \frac{[OUT_{bound}]}{1 + km_{OUTrelease,\,TEV,\,FW} \cdot [OUT_{bound}]}$$

$$v_9 = k_{OUTrelease,\,3CPRO,\,FW} \cdot [3CPRO] \frac{[OUT_{bound}]}{1 + km_{OUTrelease,\,3CPRO,\,FW} \cdot [OUT_{bound}]}$$

$$v_{10} = k_{OUTrelease,\,TEV,\,FW} \cdot [TEV] \frac{[OUT_{boundTCS}]}{1 + km_{OUTrelease.\,TEV,\,FW} \cdot [OUT_{boundTCS}]}$$

$$v_{11} = k_{OUTrelease,\,3CPRO,\,FW} \cdot [3CPRO] \frac{[OUT_{bound3CS}]}{1 + km_{OUTrelease,\,3CPRO,\,FW} \cdot [OUT_{bound3CS}]}$$

$$v_{12} = k_{OUTrelease,\,basal,\,FW} \cdot [OUT_{boundTCS}]$$

$$v_{13} = k_{OUTrelease,\,basal,\,FW} \cdot [OUT_{bound3CS}]$$

C

$$\frac{d}{dt}[3CPRO_{bound}] = -v_1 - v_2$$

$$\frac{d}{dt}[3CPRO] = +v_1 + v_2 - v_3$$

$$\frac{d}{dt}[Casp3_{bound}] = -v_4$$

$$\frac{d}{dt}[Casp3] = +v_4 - v_5$$

$$\frac{d}{dt}[Casp3_{active}] = +v_5$$

$$\frac{d}{dt}[TEV_{bound}] = -v_6$$

$$\frac{d}{dt}[TEV] = +v_6 - v_7$$

$$\frac{d}{dt}[OUT_{bound}] = -v_8 - v_9$$

$$\frac{d}{dt}[OUT_{bound3CS}] = +v_8 - v_{11} - v_{13}$$

$$\frac{d}{dt}[OUT_{boundTCS}] = +v_9 - v_{10} - v_{12}$$

$$\frac{d}{dt}[OUT] = +v_{10} + v_{11} + v_{12} + v_{13}$$

Figure S3. Mathematical model of the feedforward system. A) Model scheme. The basal and BoNT/A-LC-mediated release of 3CPRO is indicated by arrows v1 and v2, respectively. Free 3CPRO releases and activates Casp3 (v4, v5), which in turn mediates the release of TEV (v6). Released 3CPRO and TEV cleave the mCherry (OUT) crosslinker of the output material (v8-v11), leading to release of mCherry. Basal release of OUT bound via only one linker can be observed on a very slow time scale (v12-v13). The decrease of proteolytic activity of 3CPRO and TEV is indicated by arrows v3 and v7. B) Equations describing the reactions indicated in A. C) Overview of the ordinary differential equations (ODEs) describing the feedforward model.

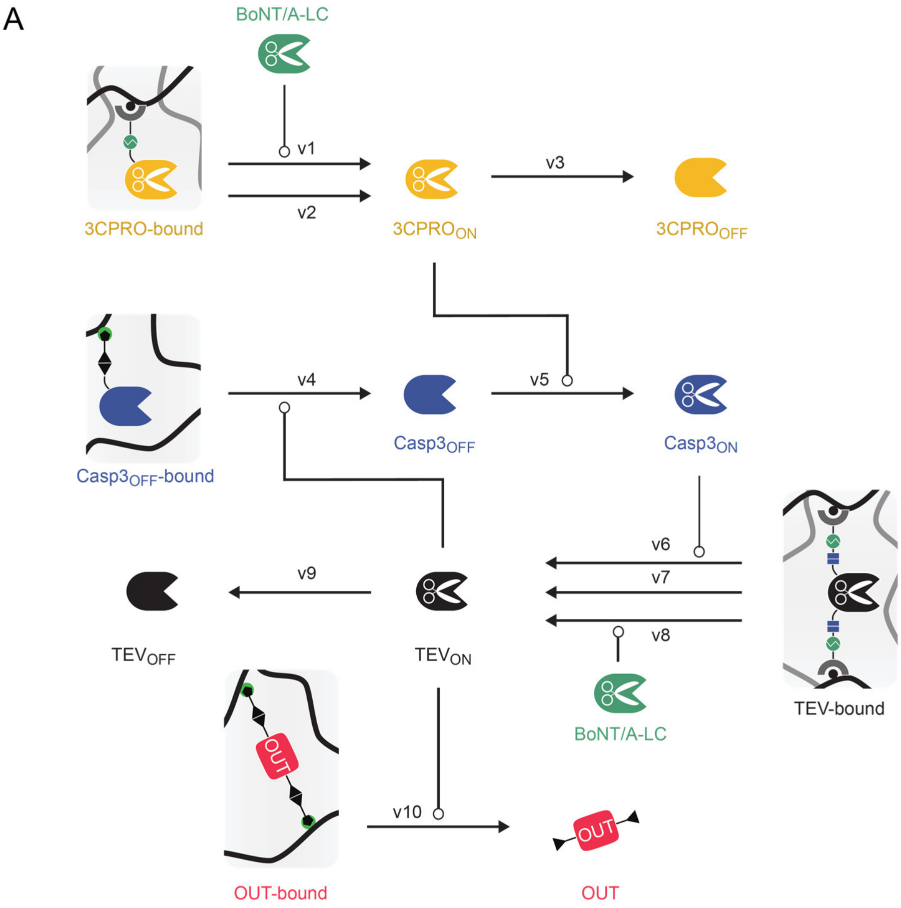

B

$$v_1 = k_{3CPROrelease,\,BoNT} \cdot [BoNT] \frac{[3CPRO_{bound}]}{1 + km_{3CPROrelease,\,BoNT} \cdot [3CPRO_{bound}]}$$

$$v_2 = k_{3CPROrelease,\,basal} \cdot [3CPRO_{bound}]$$

$$v_3 = k_{3CPROdeactivation} \cdot [3CPRO]$$

$$v_4 = k_{Casp3release,\,TEV,\,FB} \cdot [TEV] \frac{[Casp3_{bound}]}{1 + km_{Casp3release,\,TEV,\,FB} \cdot [Casp3_{bound}]}$$

$$v_5 = k_{Casp3activation,\,3CPRO} \cdot [3CPRO] \frac{[Casp3]}{1 + km_{Casp3activation,\,3CPRO} \cdot [Casp3]}$$

$$v_6 = k_{TEVrelease,\,CASP} \cdot [Casp3_{active}] \frac{[TEV_{bound}]}{1 + km_{TEVrelease,\,CASP} \cdot [TEV_{bound}]}$$

$$v_7 = k_{TEVrelease,\,basal,\,FB} \cdot [TEV_{bound}]$$

$$v_8 = k_{TEVrelease,\,BoNT,\,FB} \cdot [BoNT] \frac{[TEV_{bound}]}{1 + km_{TEVrelease,\,BoNT,\,FB} \cdot [TEV_{bound}]}$$

$$v_9 = k_{TEVdeactivation} \cdot [TEV]$$

$$v_{10} = k_{Casp3release,\,TEV,\,FB} \cdot [TEV] \frac{[OUT_{bound}]}{1 + km_{Casp3release,\,TEV,\,FB} \cdot [OUT_{bound}]}$$

C

$$\frac{d}{dt}[3CPRO_{bound}] = -v_1 - v_2$$

$$\frac{d}{dt}[3CPRO] = +v_1 + v_2 - v_3$$

$$\frac{d}{dt}[Casp3_{bound}] = -v_4$$

$$\frac{d}{dt}[Casp3] = +v_4 - v_5$$

$$\frac{d}{dt}[Casp3_{active}] = +v_5$$

$$\frac{d}{dt}[TEV_{bound}] = -v_6 - v_7 - v_8$$

$$\frac{d}{dt}[TEV] = +v_6 + v_7 + v_8 - v_9$$

$$\frac{d}{dt}[OUT_{bound}] = -v_{10}$$

$$\frac{d}{dt}[OUT] = +v_{10}$$

Figure S4. Mathematical model of the feedback system. A) Model scheme. The BoNT/A-LC-mediated and basal release of 3CPRO are indicated by arrows v1 and v2, respectively. TEV can be released by Casp3 (v6) or BoNT/A-LC (v8). The basal release of TEV is indicated by arrow v7. Free TEV triggers the release of the output mCherry (v10) and of $Casp3_{OFF}$ (v4). Released $Casp3_{OFF}$ can be activated by 3CPRO (v5). The decrease of proteolytic activity over time of 3CPRO and TEV is indicated by arrows v3 and v9, respectively. B) Equations describing the reactions indicated in A. C) Overview of the ordinary differential equations (ODEs) of the feedback model.

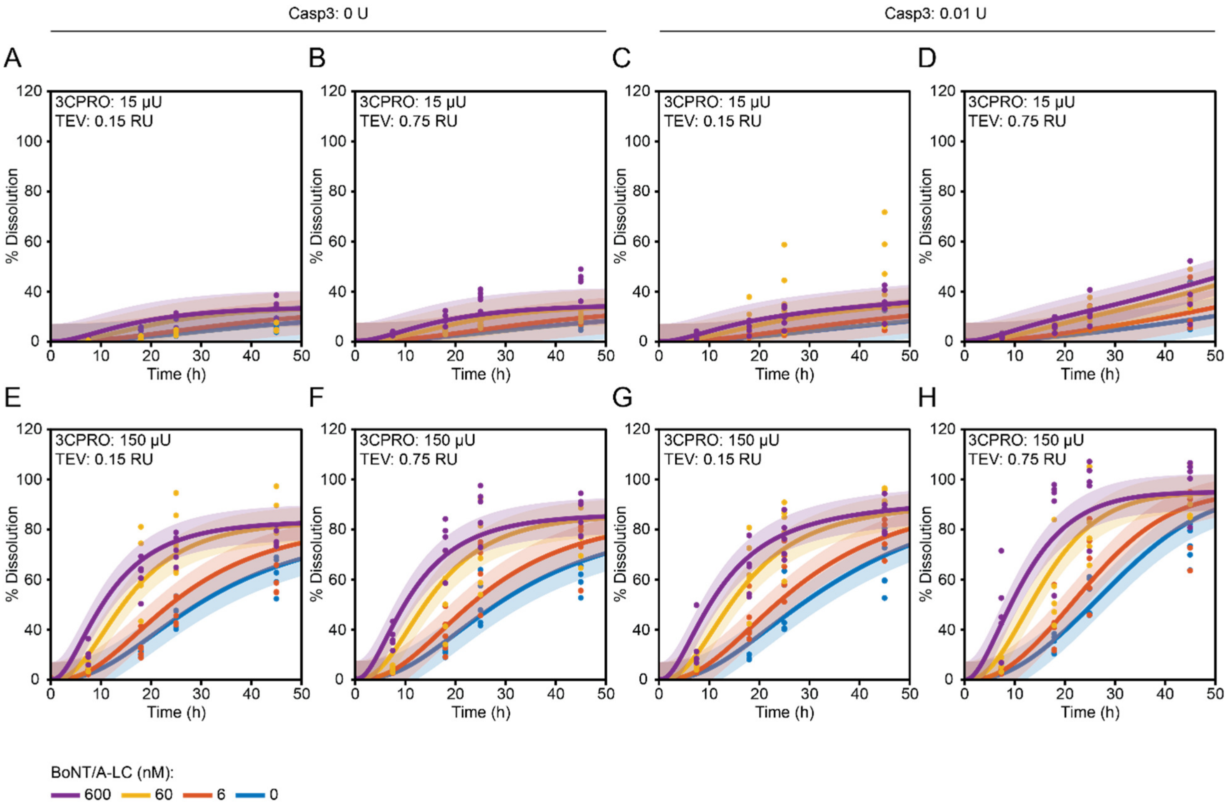

Figure S5. Characterization of the feedforward system. The system was assembled with the indicated concentrations of 3CPRO, TEV and Casp3. 0, 6, 60 and 600 nM BoNT/A-LC were added and the dissolution of the output material was monitored by quantifying the release of the output (mCherry). A-D) Feedforward system with 15 µU 3CPRO. E-H) Feedforward system with 150 µU 3CPRO. Panels A, C, E, and G correspond to systems with 0.15 RU TEV. B, D, F, and H contained 0.75 RU TEV. A, B, E, and F were synthesized without Casp3; C, D, G, and H were assembled with 0.01 U Casp3. The curves represent the feedforward model fits. The shaded bands correspond to one standard deviation.

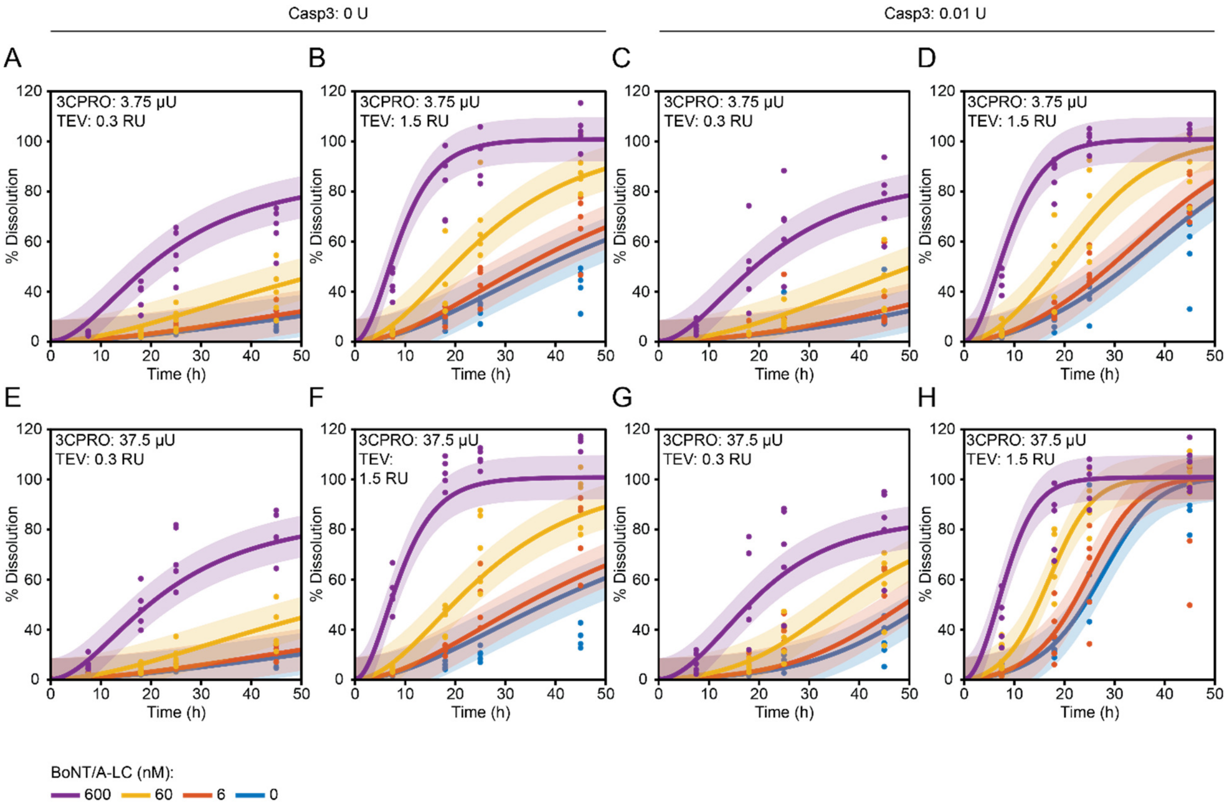

Figure S6. Characterization of the feedback system. The system was assembled with the indicated concentrations of 3CPRO, TEV and Casp3. 0, 6, 60 and 600 nM BoNT/A-LC were added and the dissolution of the output material was monitored by quantifying the release of the output (mCherry). A-D) Feedback system with 3.75 µU 3CPRO. E-H) Feedforward system with 37.5 µU 3CPRO. Panels A, C, E, and G correspond to systems with 0.3 RU TEV. B, D, F, and H contained 1.5 RU TEV. A, B, E, and F were synthesized without Casp3; C, D, G, and H were assembled with 0.01 U Casp3. The curves represent the feedback model fits. The shaded bands correspond to one standard deviation.

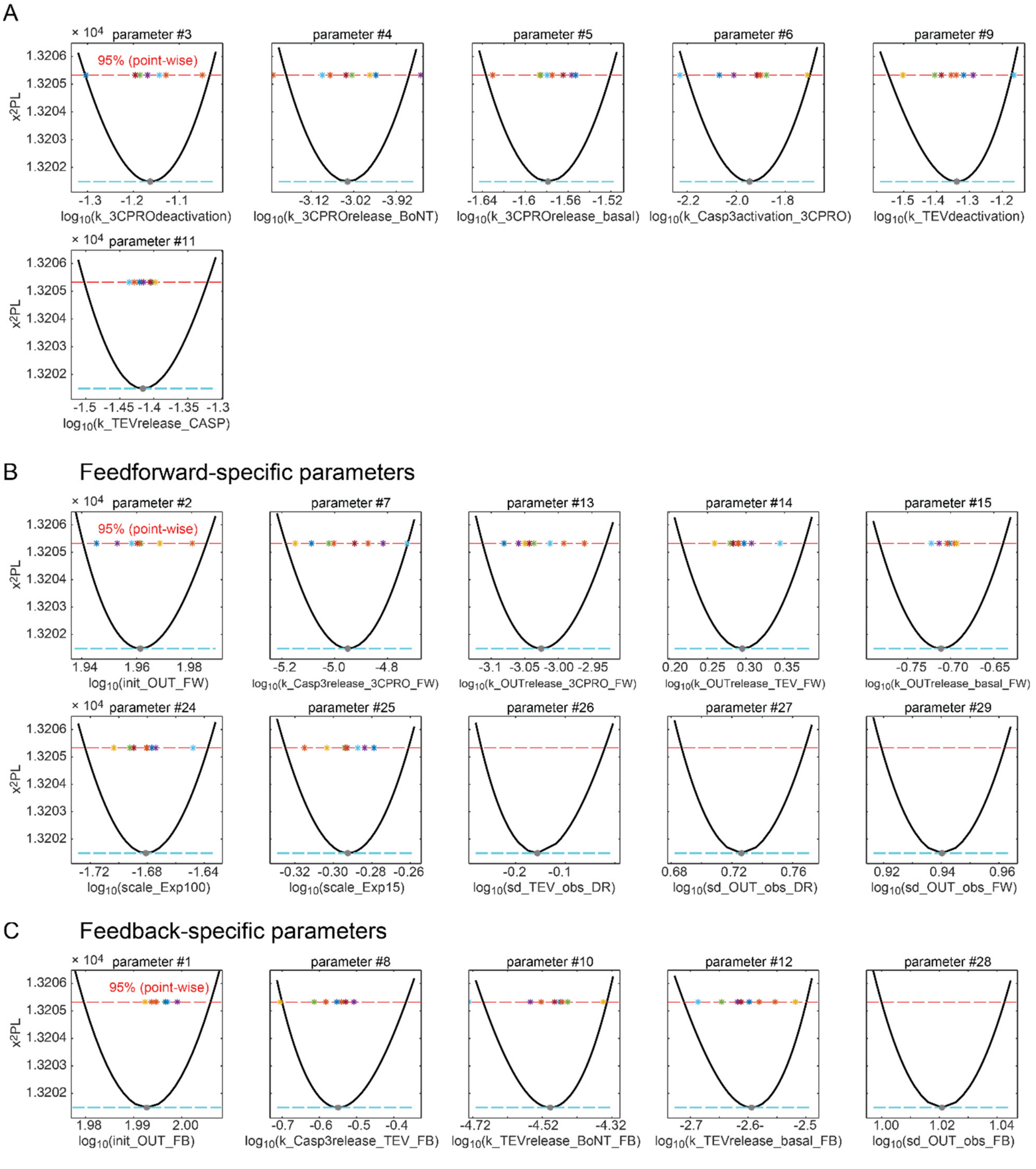

Figure S7. Profile likelihood and cross validations of estimated parameters. A) Profile likelihoods of estimated parameters of the feedforward and feedback system. B) Feedforward-specific parameters. C) Feedback-specific parameters. The profile likelihood is indicated by solid lines. The optimal parameter set is marked by a grey dot and the corresponding -2log(PL) value is indicated by the blue dashed line. The 95 % confidence level is marked by the red dashed line. Parameter values predicted by an 8-fold cross-validation are indicated by colored stars. The parameter axis is on the logarithmic scale.

Table S1. Parameters of the feedforward and the feedback system. Values are estimated by the maximum likelihood method. σ - and σ + show the 95 % point-wise confidence intervals calculated by the profile likelihood method.

Parameter	θ_{opt}	σ^-	σ^+	Unit
$K_{3CPROdeactivation}$	$6.9 \cdot 10^{-02}$	$5.0 \cdot 10^{-02}$	$9.3 \cdot 10^{-02}$	h^{-1}
$k_{3CPROrelease\_BoNT}$	$9.0 \cdot 10^{-04}$	$6.6 \cdot 10^{-04}$	$1.3 \cdot 10^{-03}$	$nM^{-1} \cdot h^{-1}$
$k_{3CPROrelease\_basal}$	$2.6 \cdot 10^{-02}$	$2.3 \cdot 10^{-02}$	$3.0 \cdot 10^{-02}$	h^{-1}
$k_{Casp3activation\_3CPRO}$	$1.1 \cdot 10^{-02}$	$6.3 \cdot 10^{-03}$	$2.0 \cdot 10^{-02}$	$\mu U^{-1} \cdot h^{-1}$
$k_{TEVdeactivation}$	$4.6 \cdot 10^{-02}$	$2.8 \cdot 10^{-02}$	$6.7 \cdot 10^{-02}$	h^{-1}
$k_{TEVrelease\_CASP}$	$3.8 \cdot 10^{-02}$	$3.2 \cdot 10^{-02}$	$4.8 \cdot 10^{-02}$	$mU^{-1} \cdot h^{-1}$
$km_{Casp3activation\_3CPRO}$	$1.7 \cdot 10^{+00}$	$2.1 \cdot 10^{+01}$	$7.32 \cdot 10^{+00}$	mU^{-1}
$k_{Casp3release\_3CPRO, FW}$	$1.1 \cdot 10^{-05}$	$6.5 \cdot 10^{-06}$	$1.9 \cdot 10^{-05}$	$\mu U^{-1} \cdot h^{-1}$
$k_{OUTrelease\_3CPRO, FW}$	$9.4 \cdot 10^{-04}$	$7.7 \cdot 10^{-04}$	$1.2 \cdot 10^{-03}$	$\mu U^{-1} \cdot h^{-1}$
$K_{OUTrelease\_TEV, FW}$	$2.0 \cdot 10^{+00}$	$1.6 \cdot 10^{+00}$	$2.4 \cdot 10^{+00}$	$RU^{-1} \cdot h^{-1}$
$K_{OUTrelease\_basal, FW}$	$1.9 \cdot 10^{-01}$	$1.7 \cdot 10^{-01}$	$2.3 \cdot 10^{-01}$	h^{-1}
$k_{Casp3release\_TEV, FB}$	$2.8 \cdot 10^{-01}$	$2.0 \cdot 10^{-01}$	$4.3 \cdot 10^{-01}$	$RU^{-1} \cdot h^{-1}$
$k_{TEVrelease\_BoNT, FB}$	$3.2 \cdot 10^{-05}$	$2.0 \cdot 10^{-05}$	$4.6 \cdot 10^{-05}$	$nM^{-1} \cdot h^{-1}$
$k_{TEVrelease\_basal, FB}$	$2.5 \cdot 10^{-03}$	$1.9 \cdot 10^{-03}$	$3.2 \cdot 10^{-03}$	h^{-1}
$Init_{OUT, FW}$	$9.1 \cdot 10^{+01}$	$8.7 \cdot 10^{+01}$	$9.6 \cdot 10^{+01}$	%
$Init_{OUT, FB}$	$9.8 \cdot 10^{+01}$	$9.5 \cdot 10^{+01}$	$1.0 \cdot 10^{+02}$	%
$Sd_{OUT\_obs, FW}$	$8.7 \cdot 10^{+00}$	$8.3 \cdot 10^{+00}$	$9.2 \cdot 10^{+00}$	%
$Sd_{OUT\_obs, FB}$	$1.0 \cdot 10^{+01}$	$9.9 \cdot 10^{+00}$	$1.1 \cdot 10^{+01}$	%
$Sd_{OUT\_obs, DR}$	$5.3 \cdot 10^{+00}$	$4.9 \cdot 10^{+00}$	$5.9 \cdot 10^{+00}$	%
$sd_{TEV\_obs, DR}$	$7.0 \cdot 10^{-01}$	$5.4 \cdot 10^{-01}$	$9.5 \cdot 10^{-01}$	%
$scale_{Exp15}$	$5.1 \cdot 10^{-01}$	$4.7 \cdot 10^{-01}$	$5.5 \cdot 10^{-01}$	1
$scale_{Exp100}$	$2.1 \cdot 10^{-02}$	$1.9 \cdot 10^{-02}$	$2.3 \cdot 10^{-02}$	1

Table S2. Plasmids used in this study.

Plasmid	Description	Reference	Used for
pHJW2	P_{T7}-His_6-TCS-mCherry-TCS-His_6 Bacterial expression vector encoding the crosslinking OUT protein of the output hydrogel of the feedback system.	[1,7]	Crosslinker of madule O of the feedback system.
pHJW4	P_{T7}-His_6-3CPRO Bacterial expression vector encoding His-tagged human rhinovirus type 14 3C protease.	[1,7]	Characterization of 3CPRO-mediated dissolution of module O of the feedforward system (Figure 4C).
pHJW14	P_{T7}-His_6-AviTag-GyrB-GyrB-CCS-BTS-TEV Bacterial expression vector encoding TEV protease coupled to tandem GyrB via a Casp3- and BoNT/A-LC-cleavable linker. The BTS is composed of the sequence encoding residues 141-206 of the SNAP25 protein. The construct was tagged with an AviTag and a hexa histidine.	unpublished	Cloning of pHJW125.
pHJW81	P_{T7}-His_6-AviTag-GyrB-BTS-CCS-TEV Bacterial expression vector encoding TEV protease coupled to GyrB via a BoNT/A-LC and Casp3-cleavable linker.	unpublished	Cloning of pHJW123.
pHJW123	P_{T7}-His_6-AviTag-GyrB-BTS-EGFP-3CPRO The sequence of EGFP-3CPRO and one half of the backbone was amplified from pHJW183 using oligonucleotides oHJW5 and oHJW427. The second half of the backbone plus His_6-AviTag-GyrB-BTS was amplified from pHJW81 using oligonucleotides oHJW6 and oHJW194. The two resulting fragments were assembled by Gibson cloning[8] , yielding a bacterial expression vector for the production of HRV14 3C protease fused to His-tagged GyrB, SNAP25(141-206) and EGFP.	This work	Supplementary Figure S1B. Cloning of pHJW178.
pHJW125	P_{T7}-His_6-AviTag-GyrB-GyrB-CCS-BTS*-TEV Q175N and Q178N mutations were introduced into BTS via site-directed mutagenesis of pHJW14 using oligonucleotides oHJW486 and oHJW487. This resulted in a bacterial expression vector encoding TEV protease linked to tandem GyrB via a CCS and BTS* (SNAP25(141-206, Q175N, Q178N))-containing linker.	This work	Characterization of TEV-mediated dissolution of material O of both systems (Figure 4).
pHJW144	P_{T7}-His_6-Stag-BoNT/A-LC(8-415)-His_6 The BoNT/A-LC(8-415) sequence was amplified using oligonucleotides oHJW416 and oHJW417. BoNT/A-LC was a gift from Axel Brunger (Addgene plasmid #31602).[9] The plasmid backbone[10] was amplified using oligonucleotides oHJW418 and oHJW419. The two fragments were assembled by Gibson cloning, resulting in a bacterial expression vector encoding His- and S-tagged light chain of the botulinum neurotoxin A.	This work	Protease domain of BoNTA inducing the materials systems.
pHJW178	P_{T7}-His_6-AviTag-GyrB-SNAP25(141-206,Q175N,Q178N)-GFP-3CPRO Q175N and Q178N mutations were introduced into pHJW123 by site-directed mutagenesis using oligonucleotides oHJW486 and oHJW487. This resulted in a bacterial expression vector encoding the 3CPRO fusion construct of the receiver module R.	This work	3CPRO construct of module R of the feedforward and feedback system.
pHJW181	P_{T7}-Casp3($3CPRO_{ind}$)-TCS-His_6 Bacterial expression vector encoding 3CPRO-inducible $Casp3_{OFF}$ of the feedback system.	[1,7]	3CPRO-inducible $Casp3_{OFF}$ of the feedback system.

Plasmid	Description	Reference	Used for
pHJW182	P_{T7}-Casp3(3CPRO$_{ind}$)-3CS-His$_6$<br>The TCS of pHJW181 was exchanged with 3CS by amplifying Casp3(3CPRO$_{ind}$) together with one half of the backbone using oligonucleotides oHJW6 and oHJW502, and the 3CS-His-tag containing linker region plus the second part of the backbone using oligonucleotides oHJW5 and oHJW501. The two resulting fragments were assembled by Gibson cloning, yielding a bacterial expression vector for Casp3$_{OFF}$ of the feedforward system.	This work	3CPRO-inducible Casp3$_{OFF}$ of the feedforward system.
pHJW183	P_{T7}-His$_6$-GyrB-mEGFP-3CPRO<br>Bacterial expression vector encoding the human rhinovirus type 14 3C protease fused to His-tagged bacterial gyrase subunit B and EGFP.	[1,7]	Supplementary Figure S1A. Cloning of pHJW123.
pHJW199	P_{T7}-His$_6$-GyrB-CCS-TEV-CCS-GyrB<br>Bacterial expression vector encoding the TEV construct of module T2 of the feedforward system.	[1,7]	Casp3-cleavable TEV construct of the feedforward system.
pHJW261	P_{T7}-His$_6$-TCS-mCherry-3CS-His$_6$<br>The 3CS-His-linker was amplified from pKJ60 using oligonucleotides oHJW12 and oHJW525. The His-TCS-linker was amplified from pKJ60 using oligonucleotides oHJW7 and oHJW8. mCherry was amplified from pKJ10 using oligonucleotides oHJW9 and oHJW10. The two halves of the backbone were amplified from pKJ60 using oligonucleotides oHJW1 and oHJW5, and oHJW6 and oHJW13. The resulting fragments were assembled by Gibson cloning, yielding a bacterial expression vector encoding the hydrogel crosslinker of material O of the feedforward system.	This work	Crosslinker of material O of the feedforward system.
pHJW265	P_{T7}-His$_6$-GyrB-BTS*-CCS-TEV-CCS-BTS*-GyrB-His$_6$<br>The sequence of the backbone plus His-tagged GyrB was amplified from pHJW183 using oligonucleotides oHJW124 and oHJW192. The BTS* (SNAP25(141-206,Q175N,Q178N))-CCS sequence was amplified from pHJW178 using oligonucleotides oHJW193 and oHJW194. TEV was amplified from pHJW14 using oligonucleotides oHJW184 and oHJW195. CCS-BTS* linker was amplified from pHJW178 using oligonucleotides oHJW189 and oHJW194. GyrB-His-tag was amplified from pHJW183 using oligonucleotides oHJW582 and oHJW583. The BTS*-CCS and TEV fragment were assembled by fusion PCR using oligonucleotides oHJW184 and oHJW193. The resulting fusion PCR product and the remaining PCR products were assembled by Gibson cloning, yielding a bacterial expression vector encoding the TEV construct of module T2 of the feedback system.	This work	Casp3- and BoNT/A-LC-cleavable TEV construct of the feedback system.
pKJ10	P_{T7}-His$_6$-mCherry<br>Bacterial expression vector encoding hexahistidine-tagged mCherry.	Unpublished	Cloning of pHJW261.
pKJ60	P_{T7}-His$_6$-TCS-FM<br>Bacterial expression vector encoding the F36M variant of human FK-binding protein 12 (FM) linked to a TEV-removable His-tag.	unpublished	Cloning of pHJW261.

Table S3. Primers used for cloning of the plasmids in Table S2. Annealing sequences are underlined.

Oligo	Sequence (5'->3')	Reference
oHJW1	CTTGATCCGGCTGCTAACAAAG	[1,7]
oHJW5	CTTTGATCTTTTCTACGGGGTCTG	[1,7]
oHJW6	GCGTCAGACCCCGTAGAAAAG	[1,7]
oHJW7	GAGACCACAACGGTTTCCCTC	[1,7]
oHJW8	GTTATCCTCCTCGCCCTTGCTCACGAATTCACCGGTACGCGTAGAAC	[1,7]
oHJW9	GTGAGCAAGGGCGAGGAG	[1,7]
oHJW10	CTTGTACAGCTCGTCCATGCC	[1,7]
oHJW12	CTTTGTTAGCAGCCGGATCAAGCTTTTAATGGTGATGGTGATGATGGAATTCACCGGTACGCGTAGAAC	[1,7]
oHJW13	CTAGAGGGAAACCGTTGTGGTC	[1,7]
oHJW124	TAAAAGCTTGATCCGGCTGCTAAC	This work
oHJW184	GCGACGGCGACGACGATTC	This work
oHJW189	GAATCGTCGTCGCCGTCGCGGCTCTGGCTCTGATGAAGTGGATGCGCGCGAAAACGAAATGG	This work
oHJW192	CGTTTTCGCGCGCAGAGCCAGAGCCGGGGCCTTCATAGTG	This work
oHJW193	CTCTGCGCGCGAAAACG	This work
oHJW194	GCCAGAGCCGCCGGATCCGCCGCCGCTGCCCAGCATTTTG	This work
oHJW195	GGCGGATCCGGCGGCTCTGGCGATGAAGTGGATGGCACCAGC	This work
oHJW416	CGACGACAAGGCCATGGCTCCGTTTGTGAACAAACAGTTTAACTATAAAGATCCTGTAAATGGTGTTGATATTGC	This work
oHJW417	CGAGTTAATGGTGATGGTGATGATGAAATTCAAACAGGCCGGTAAAGTTTTTTAGTTTAGTAAAATTCATATTATTAATTTCT GTATTTTGACC	This work
oHJW418	CATCATCACCATCACCATTAACTCG	This work
oHJW419	AGCCATGGCCTTGTCGTC	This work
oHJW427	GGATCCGGCGGCTCTGGCATGGTGAGCAAGGGCGAG	[1,7]
oHJW486	GATATGGGCAACGAAATTGATACCAACAACCGCAACATTGATCGCATTATGGAAAAAGCG	This work
oHJW487	CGCTTTTTCCATAATGCGATCAATGTTGCGGTTGTTGGTATCAATTTCGTTGCCCATATC	This work
oHJW501	CTGGAAGTGCTGTTTCAGGGCCCCGGAGGTGGACCCGCC	This work
oHJW502	GGGCCCTGAAACAGCACTTCCAGACCGCCACCGCCGCTGCCGCCGCCAGAATAAAAATAGAGTTC	This work
oHJW525	GCATGGACGAGCTGTACAAGTCTGGCGGCGGCAGCGGCGGTGGCGGTCTGGAAGTGCTGTTTCAGGGCCCCGGAGGTGGA CCCGCC	This work
oHJW582	GCGGATCCGGCGGCTCTGGCTCGAATTCTTATGACTCCTCCAGTATC	This work
oHJW583	GCAGCCGGATCAAGCTTTTAATGATGGTGATGATGATGGCCTTCATAGTGGAAGTGGTCTTC	This work

References

[1] H. J. Wagner, R. Engesser, K. Ermes, C. Geraths, J. Timmer, W. Weber, *Mater. Today* **2018**, in press.

[2] A. Raue, C. Kreutz, T. Maiwald, J. Bachmann, M. Schilling, U. Klingmüller, J. Timmer, *Bioinformatics* **2009**, *25*, 1923.

[3] T. Maiwald, H. Hass, B. Steiert, J. Vanlier, R. Engesser, A. Raue, F. Kipkeew, H. H. Bock, D. Kaschek, C. Kreutz, J. Timmer, *PLoS One* **2016**, *11*, e0162366.

[4] A. Raue, B. Steiert, M. Schelker, C. Kreutz, T. Maiwald, H. Hass, J. Vanlier, C. Tönsing, L. Adlung, R. Engesser, W. Mader, T. Heinemann, J. Hasenauer, M. Schilling, T. Höfer, E. Klipp, F. Theis, U. Klingmüller, B. Schöberl, J. Timmer, *Bioinformatics* **2014**, *31*, 3558.

[5] A. C. Hindmarsh, P. N. Brown, K. E. Grant, S. L. Lee, R. Serban, D. E. Shumaker, C. S. Woodward, *ACM Trans. Math. Softw.* **2005**, *31*, 363.

[6] T. F. Coleman, Y. Li, *SIAM J. Optim.* **1996**, *6*, 418.

[7] H. J. Wagner, R. Engesser, K. Ermes, C. Geraths, J. Timmer, W. Weber, *Data in Brief* **2018**, *19*, 665.

[8] D. G. Gibson, L. Young, R. Chuang, J. C. Venter, C. A. Hutchison, H. O. Smith, *Nat. Methods* **2009**, *6*, 343.

[9] J. E. Zuniga, J. J. Schmidt, T. Fenn, J. C. Burnett, D. Araç, R. Gussio, R. G. Stafford, S. S. Badie, S. Bavari, A. T. Brunger, *Structure* **2008**, *16*, 1588.

[10] A. H. Zisch, U. Schenk, J. C. Schense, S. E. Sakiyama-Elbert, J. A. Hubbell, *J. Control. Release* **2001**, *72*, 101.
